# Development of an iPSC-derived tissue-resident macrophage-based platform for the in vitro immunocompatibility assessment of human tissue engineered matrices

**DOI:** 10.1038/s41598-024-62745-1

**Published:** 2024-05-28

**Authors:** Nikolaos Poulis, Marcy Martin, Simon P. Hoerstrup, Maximilian Y. Emmert, Emanuela S. Fioretta

**Affiliations:** 1 Institute for Regenerative Medicine (IREM), University of Zurich, Wagistrasse 12, 8952 Schlieren, Switzerland; 2 Wyss Zurich, University and ETH Zurich, Zurich, Switzerland; 3 Department of Cardiothoracic and Vascular Surgery, Deutsches Herzzentrum der Charité (DHZC), Berlin, Germany; 4 Charité – Universitätsmedizin Berlin, Corporate Member of Freie Universität Berlin and Humboldt-Universität zu Berlin, Berlin, Germany; 5 Institut für Regenerative Medizin (IREM), University of Zurich, Moussonstrasse 13, 8044 Zurich, Switzerland

**Keywords:** Resident tissue macrophages, Induced pluripotent stem cells, iPSC-derived macrophages, Tissue culture, Extracellular matrix, Mass spectroscopy, Induced pluripotent stem cells, Tissues

## Abstract

Upon implanting tissue-engineered heart valves (TEHVs), blood-derived macrophages are believed to orchestrate the remodeling process. They initiate the immune response and mediate the remodeling of the TEHV, essential for the valve’s functionality. The exact role of another macrophage type, the tissue-resident macrophages (TRMs), has not been yet elucidated even though they maintain the homeostasis of native tissues. Here, we characterized the response of hTRM-like cells in contact with a human tissue engineered matrix (hTEM). HTEMs comprised intracellular peptides with potentially immunogenic properties in their ECM proteome. Human iPSC-derived macrophages (iMφs) could represent hTRM-like cells in vitro and circumvent the scarcity of human donor material. iMφs were derived and after stimulation they demonstrated polarization towards non-/inflammatory states. Next, they responded with increased IL-6/IL-1β secretion in separate 3/7-day cultures with longer production-time-hTEMs. We demonstrated that iMφs are a potential model for TRM-like cells for the assessment of hTEM immunocompatibility. They adopt distinct pro- and anti-inflammatory phenotypes, and both IL-6 and IL-1β secretion depends on hTEM composition. IL-6 provided the highest sensitivity to measure iMφs pro-inflammatory response. This platform could facilitate the in vitro immunocompatibility assessment of hTEMs and thereby showcase a potential way to achieve safer clinical translation of TEHVs.

## Introduction

Valvular heart disease is a major cause of morbidity and mortality worldwide, and the number of patients requiring heart valve replacements is increasing every year^1^. In situ heart valve tissue engineering aims to provide durable heart valve replacements with repair and remodeling capacity^2–5^. As one strategy, human tissue engineered matrices (hTEMs) have been proposed as a promising solution for the manufacturing of TEHVs with self-repair and adaptive remodeling potential, for either pulmonary^6,7^ or aortic^8^ applications. Importantly, hTEM properties and composition shift throughout culture times and production^9^. To enable off-the-shelf availability and improve immunocompatibility, hTEMs undergo a decellularization process while maintaining the ECM composition of the implant^9,10^. Upon implantation of a hTEM-based TEHV, monocytes and macrophages, as the first line of defense, are attracted to the tissue engineered implant and respond through an inflammatory cascade^11^. Within in situ tissue engineering, macrophages have been identified as key players in the initial stages of tissue remodeling^12,13^. As macrophages infiltrate the implanted foreign material, it is hypothesized that by their initial pro-inflammatory activation is key to the adaptive remodeling cascade^4,14^. Subsequently, the initial macrophage pro-inflammatory response is resolved when stimulants that caused the response are removed, which is then followed by macrophage polarization towards an anti-inflammatory state^4,11^. During the initial steps of tissue remodeling, macrophages can respond to stimulants such as implant components (extracellular matrix (ECM) proteins and synthetic polymers), by polarizing to either a pro- or an anti-inflammatory phenotype and release inflammatory cytokines the promote the cell recruitment stages that follow^15–19^. Several in vitro studies have assessed the role of ECM proteins in regulating macrophage polarization^15–20^. The cells of choice for these in vitro experiments have most often been peripheral blood-derived macrophages as they are easily accessible and readily available^16,19,21^. However, peripheral blood-derived macrophages introduce an inherent limitation to the investigation of immunocompatibility due to donor-to-donor variability, which can impose a variation of immune responses^21^.

Tissue-resident macrophages (TRMs) are a subset of macrophages that originate from embryonic primitive transient (c-Myb^−^) hematopoiesis, different from peripheral blood-derived macrophages^22^. In adulthood, TRM function is linked with organ tissue homeostasis, immune surveillance, and tissue repair^23,24^. Therefore, the use of TRMs for immunocompatibility assessments would be preferable. However, the paucity of such cells and the very limited donor material for the isolation of significant cell numbers makes it difficult to investigate their mechanisms in tissue remodeling^22,24–26^. Hence, there is no compelling evidence from in vitro immunocompatibility platforms that focus on assessing the role of TRMs in tissue remodeling as of yet, a role that cannot be represented by peripheral blood-derived macrophages^25,27–29^.

Although there is a lack of primary TRMs, human iPSC-derived macrophages (iMφs) enable scalable TRM-like cell production in vitro^12,28–31^. iMφs have been proven to share common embryonic primitive transient (c-Myb^−^) hematopoietic origins with native TRMs, and share similar polarization capacity towards pro- and anti-inflammatory phenotypes^31,32^. In fact, as described in previous study by Gutbier et al., there is high commonality in polarization capacity between iMφs and PBMC-derived macrophages. However, when sensitivity was assessed, iMφs showed higher sensitivity in polarization toward pro-and anti-inflammatory conditions, whereas the PBMC-derived macrophages showed bias toward pro-inflammatory. Therefore, iMφs have been proposed as a valid model for TRM-like cells. Additionally, iMφs overcome the TRM scarcity with in vitro scalability as well as the inter-donor variability of peripheral blood-derived macrophages^30–32^.

In this study, we aimed to use iMφs as a potential cell source for the development of an in vitro immunocompatibility assessment platform. To achieve this, we first established iMφ-precursor production and differentiation into iMφs. Then, we performed an in-depth characterization using biological markers and functionally assessed iMφs polarization potential. Finally, in a proof-of-concept experiment, we evaluated the capacity of iMφs to adopt pro- or anti-inflammatory phenotypes in response to hTEM composition. In the previously performed in-depth analysis, the proteome of these hTEMs indicated a number of intracellular peptides, some with potentially immunogenic (ACTA2, HSPB1, HSPA8) and xenogenic (bovine FGB, HBA1) properties, of which could potentially contribute to pro- or anti-inflammatory responses. Here, we aimed to identify if ECM maturation in combination with determined proteomic composition could influence iMφ polarization in vitro, and hence be used as a functional test for TRM-like cell assessment for their capacity to polarize in vitro.

## Materials and methods

### Study layout

In this study, iMφs were used as a platform to assess the immunogenicity of hTEMs in vitro. Initially, iMφs were differentiated from iPSCs according to established protocols^30,31^ that were adapted as described in “iMφ derivation” section, and their polarization potential was evaluated (“iMφ polarization” section). Finally, the immunocompatibility assessment was performed (“Immunocompatibility assessment” section). To assess whether iMφs were functionally significant and can show sensitivity to distinct substrates, we exposed iMφs to hTEM samples in culture for 3 and 7 days. hTEM samples were comprised of three different time points of 2-week (2W), 4-week (4W), and 6-week (6W) of tissue culture times. hTEMs were characterized with mass spectroscopy to identify their proteomic composition, with a focus on the presence of potential immunogenic proteins still retained after decellularization. After co-culturing iMφs for 3 and 7 days, flow cytometry and ELISA were performed to assess the cellular polarization capacity towards pro- and anti-inflammatory conditions with the corresponding cytokine release.

### iMφ derivation

#### iPSC culture

The iPSC line WTB6 (RRID:CVCL_VM30), previously published from Miyaoka et al.^33^, was obtained from a healthy female donor using the episomal reprogramming method^34^. Informed consent was obtained for this procedure as stated in the original publication^33^. All experiments have been conducted under the license A141376-03 approved by the Federal Coordination Centre for Biotechnology. All iPSCs were maintained and expanded on Matrigel (Corning) coated six well plates using mTeSR1 (STEMCELL) medium with daily replacement. iPSC passaging was performed by using Gentle Cell Dissociation Reagent (STEMCELL).

#### Embryoid body (EBs) formation

iPSCs were harvested and suspended into a single cell solution by using StemPro Accutase (Gibco). After harvesting, the cells were resuspended in mTeSR1 supplemented with 50 ng/mL BMP-4 (PeproTech), 50 ng/mL VEGF (PeproTech), 20 ng/mL SCF (PeproTech) and 10 μM ROCK inhibitor (Tocris). 4 × 10^6^/mL cells were seeded in AggreWell™ 800 (STEMCELL) and centrifuged at 100×*g* for 6 min^29,30^. To avoid bubble formation in the AggreWell™ plates, medium was placed, centrifuged at 100×*g* for 6 min, and removed prior to cell seeding. After seeding cells, the medium was replaced daily for 4 days until the EBs were formed in the bottom of the wells.

#### iMφ derivation

In order to induce non-myeloid differentiation at day 4, the formed EBs were transferred to T75 flasks in factory medium (FM) X-VIVO 15™ (Lonza) supplemented with GlutaMax 1% (Gibco), b-mercaptoethanol (Gibco), M-CSF 100 ng/mL (PeproTech) and IL-3 25 ng/mL (PeproTech). After day 14, medium was refreshed every 4 days. At day 22 onwards, iMϕ precursors were isolated and plated on 6-well plates in order to differentiate into iMφs. The differentiation medium (DM) used for the terminal differentiation is X-VIVO 15™ (Lonza) supplemented with GlutaMax 1% (Gibco), b-mercaptoethanol (Gibco), and M-CSF 100 ng/mL (PeproTech) for 7 days^30^.

#### Gene expression

RNA isolation was performed using an RNeasy Minikit (Qiagen) and cDNA was made by using iScript (BioRad). qPCR was performed using SYBR™ Green (Applied Biosystems). CT values and melt curve calculations were obtained by QuantStudio™ 7 Flex Real-Time PCR System 384 (Applied Biosystems). Primers are listed in Supplementary Table 2. The final analysis of the data was performed in GraphPad Prism (v8.0.2).

### iMφ polarization

For standardized iMϕ polarization into pro- or anti-inflammatory states, iMϕs following terminal differentiation (M0) state were plated for 72 h in DM supplemented with either LPS 100 ng/mL (Sigma) and IFN-γ 20 ng/mL (PeproTech) for pro-inflammatory polarization (M1), or IL-13 20 ng/mL (PeproTech) and IL-4 20 ng/mL (PeproTech) for anti-inflammatory polarization (M2). Pro- or anti-inflammatory polarization was measured at both gene (qPCR) and protein (flow-cytometry, ELISA) levels after 72 h.

### Immunocompatibility assessment

#### hTEM production

hTEMs (n = 12) were produced as previously described by using non-woven polyglycolic acid (PGA; Cellon) meshes coated with 1% poly-4-hydroxybutyrate (P4HB) sutured onto a nitinol stent^6,8^, then cultured using the rotation-based tissue culture (RTC) insert as previously described (Supplementary Methods)^9^.

Purified human dermal fibroblasts (hDFBs, CellSystems Biotechnologie Vertrieb GmbH (Lot#: 00896) were seeded onto the PGA-P4HB scaffolds (1 × 10^6^ cells/cm^2^) using fibrin as a cell carrier as previously described^35^. After seeding, the constructs were incubated statically in cell culture medium overnight to favour cell adhesion and then transferred to a pleated roller-bottle for tissue culture (1450 cm^2^, TufRol, Nunc). After 2W, 4W, or 6W of tissue culture, hTEMs (n = 3 per timepoint) were washed in PBS and decellularized^36^ using an optimized protocol based on the work of Lintas et al.^8^. Lastly for the hTEM/iMφs cultures, the hTEMs were sterilized and cut into10 mm diameter discs. The conditions that were used for these cultures are PGA, PGA/FIB, 2W, 4W and 6W-hTEMs for 3 and 7 days.

#### hTEM characterization

##### dsDNA quantification

hTEM decellularization efficiency was verified by quantifying double strand DNA (dsDNA). Briefly, 1–2 mg of lyophilized hTEM samples (n = 4 for each time point, in technical triplicate) were digested with papain (Worthington) in a digestion buffer solution. dsDNA analysis was performed using a Qubit™ dsDNA HS Assay kit and a Qubit 3.0 fluorometer (Thermofisher). The data was normalized to total weight of dry tissue.

##### Mass spectrometry analysis (LC–MS/MS)

Mass spectrometry analysis of the hTEM samples (5 × 5 mm^2^) was used to gain insights on the ECM proteomic composition and its evolution over tissue culture duration^9^.

hTEM samples (n = 4 for each time point) were therefore processed, analyzed, normalized, and statistically evaluated by the quality of the identified proteins by the Functional Genomic Center Zurich (Supplementary Methods). To analyse the hTEM proteome, the LC–MS/MS (Nano Acquity UPLC coupled to a Q-Exactive mass spectrometer (Thermo)) method was used. Proteins were identified and quantified using MaxQuant software and the Andromeda search engine. The detailed analysis workflow is presented in Supplementary Methods.

##### Label-free quantification

Normalized MS1intensities were used for the label-free quantification of all proteins identified in the hTEM (n = 4 for each time point). The results were presented as an average normalized MS1 value across samples with the same tissue culture time point, using a heat map (normalized MS1 value ranging from − 5 to + 11 for, respectively, for the least and most abundant proteins).

An analysis of protein fold change (FC) was calculated for proteins having a minimum of 2 identified peptides and analysed by a moderated t-test^37^ between each time point (4W vs 2W; 6W vs 2W; 6W vs 4W) (Supplementary Figs. 3–5).

Xenogenic proteins (with bovine origin), potentially immunogenic proteins (Supplementary Table 1, Supplementary Fig. 5), and the 20 most abundant intracellular proteins were investigated in this study.

#### iMϕ preparation for hTEM/iMφ cultures

M0 iMφs, following terminal differentiation, were harvested with gentle cell dissociation reagent (STEMCELL). 250,000 cells were resuspended in 20 μL of DM and were seeded on top either PGA-P4HB scaffolds, fibrin coated PGA-P4HB (PGA/FIB), or the 2W, 4W and 6W-hTEM discs in 48-well plates. iMφs polarization was assessed after 3 and 7 days of culture in a total of n = 3 separate experiments.

#### Flow cytometry

Flow cytometry was performed to assess the polarization from surface markers using the following anti-human surface marker conjugated antibodies (Table 1). iMφs were detached from plates using Gentle Cell Dissociation Reagent (STEMCELL). In the hTEM/iMφs cultures, iMφs were detached from the hTEMs or PGA-P4HB scaffolds using Gentle Cell Dissociation Reagent for 11 min at 37 °C on an orbital rocker. The solutions were centrifuged for 3 min at 300×*g*. After one PBS washing step, iMφs were stained for viability using Zombie Violet™ (BioLegend). iMφs were then fixed with 1% paraformaldehyde (Thermo) for 1 h at 4 °C. After fixation, iMφs were washed 3 times with 300 μL FACS buffer consisting of Hank’s Balanced salt solution (HBSS) (Gibco), 2% FBS, 0.2% sodium azide, 0.5 mM EDTA (UltraPure™ EDTA pH 8.0) (Invitrogen™). iMφs were then centrifuged and stained with selected surface markers mixed with FACS buffer for 1 h at 4 °C in the dark (Table 1). After washing and resuspending in 300 μL FACS buffer, iMφs were analyzed using the LSR II Fortessa™ 4L (BD, acquisition software: BD FACSDiva™ v8) and analysis with the FCS 6 Express software (version 6.06.0025, De Novo Software by Dotmatics). Compensation controls were done by using flow cytometry beads, OneComp eBeads™ (Invitrogen™). The statistical analysis of the data was performed in GraphPad Prism (v8.0.2).

**Table 1:** List of antibodies used for flowcytometry analysis.

Marker	Fluorophore	Clone	Brand
CD34 stem cells	FITC	4H11(APG)	Abcam ab18227
CD163 macrophages/anti-inflammatory macrophages	PE	REA812	Miltenyi Biotech 5200703163
CD45 macrophages	APC	REA747	Miltenyi Biotech 5200908613
CD14 macrophage precursors/macrophages	PE	TUK4	Miltenyi Biotech 5200703151
CD86 pro-inflammatory macrophages	FITC	REA968	Miltenyi Biotech 5200703165
CD197 pro-inflammatory macrophages	FITC	REA546	Miltenyi Biotech 5200607322
CD206 anti-inflammatory macrophages	PE	DCN228	Miltenyi Biotech 5200705185

#### ELISA

The supernatant was isolated from each culture condition and were kept at − 80 °C until further analysis. After thawing, the supernatants were used immediately for cytokine quantification using following kits according to the manufacturer’s instructions: Proteome Profiler Human Cytokine Array Kit for 36 cytokines (Biotechne, ARY005B). Secreted interleukins were identified using the following ELISA kits (sample dilution 1:5000): TNF-α (Invitrogen, BMS223HS), IL-6 (Invitrogen, BM213HS), IL-8 (Invitrogen, BMS204-3INST), IL-1β (Invitrogen, BMS224HS) and IL-10 (Invitrogen, BMS215HS). Quantification was analyzed performed by a microplate reader, TECAN M1000 pro (TECAN, Switzerland), software i-control™ (version: 2.0.10.0). The statistical analysis of the data was performed in GraphPad Prism (v8.0.2).

## Results

### iMφs can be derived from human pluripotent stem cells

**Figure 1:**
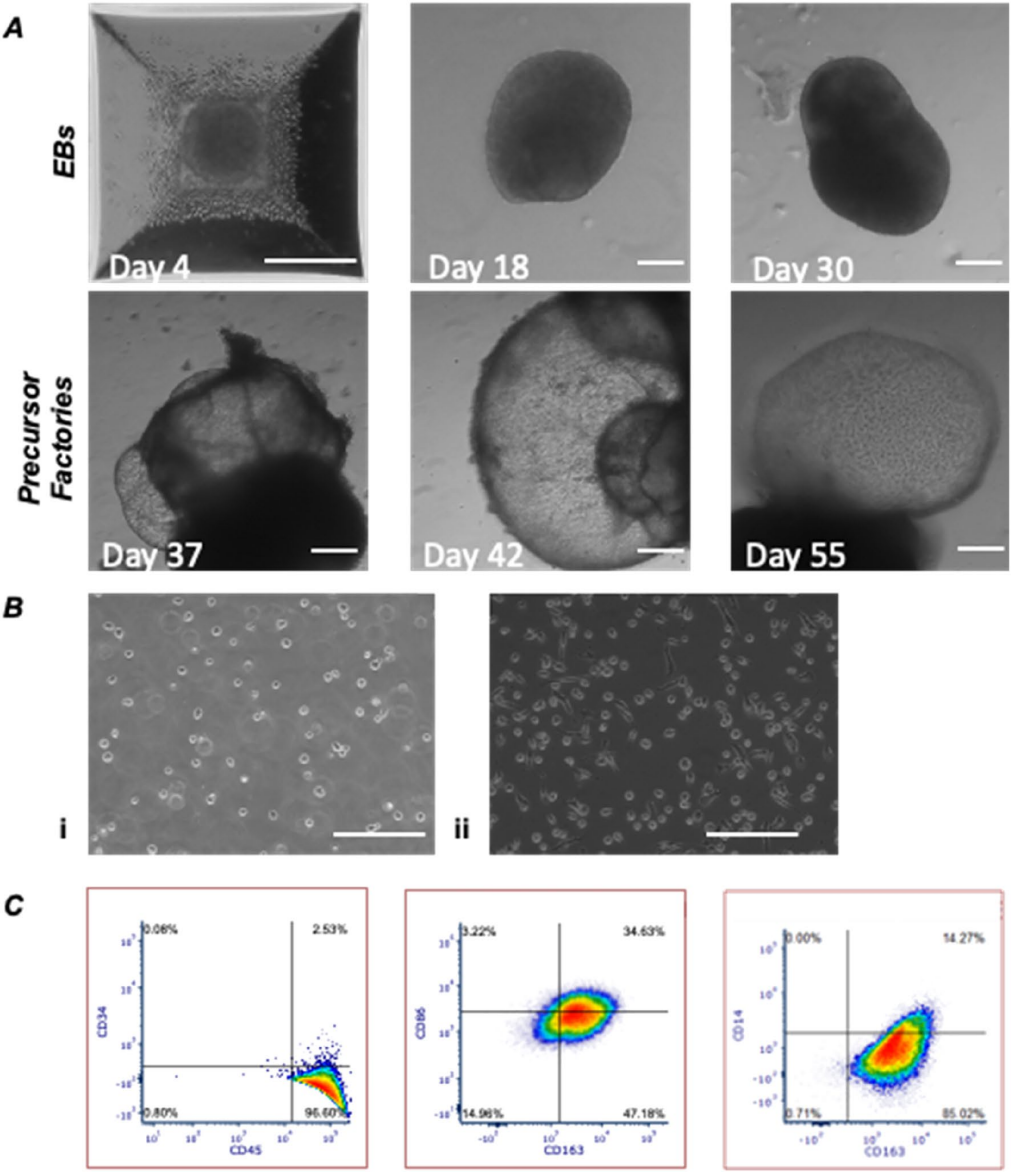
Formation of iMϕ precursor factories and characterization of differentiated iMφs. (**A**) Progression of iMφ precursor factory formation towards the yolk sac-like structures (scale bar indicates 200 μm). (**B**) Brightfield images of (**i**) iMϕ precursor showing initial non-adherence and (**ii**) iMφs after differentiation in DM medium for 7 days, which adhered to the bottom of the well. (**C**) Flow cytometry assessment of iMφs after differentiation in DM medium for 7 days (n = 3; 50,000 cell for each experiment).

**Figure 2:**
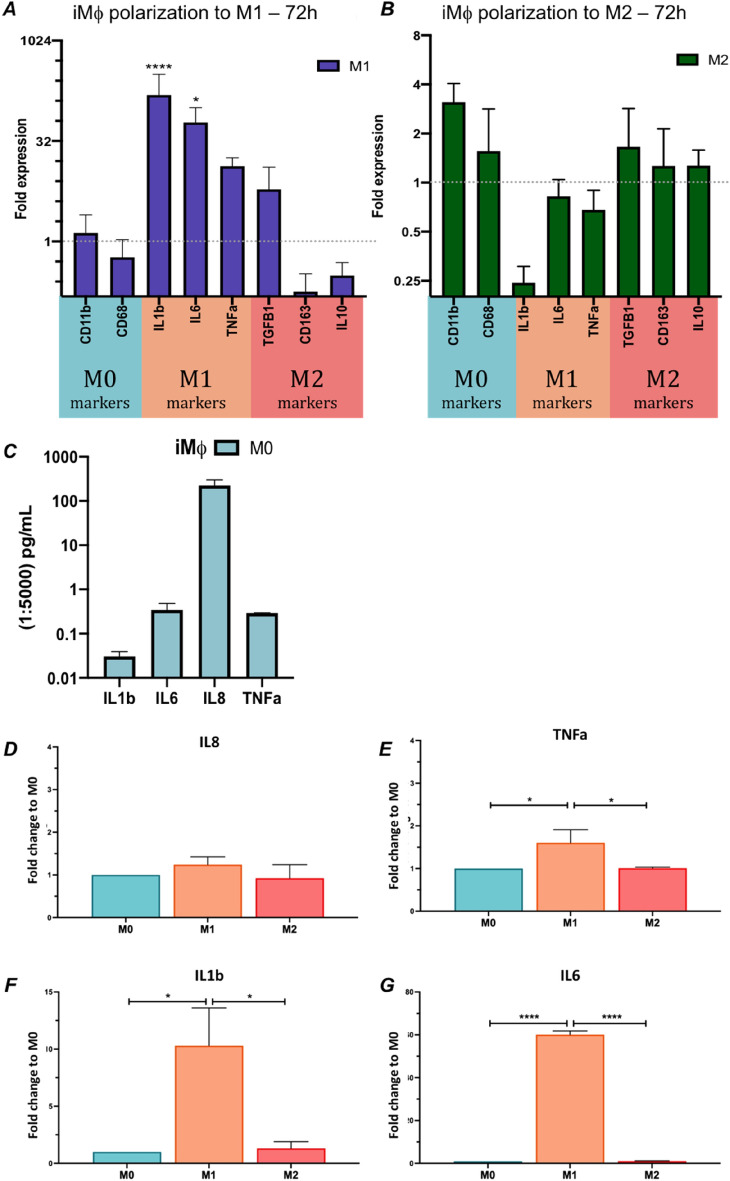
Polarization of iMφs towards pro- and anti-inflammatory states for 72 h. (**A**) Gene expression in iMφs polarized with M1 stimulation (LPS/IFN-γ) for 72 h. (**B**) Polarization of iMφs with M2 stimulation (IL-4/IL-13) for 72 h. (**C**) ELISA raw quantification of iMϕ M0 secreted interleukins after differentiation for 7 days in DM medium (dilution of 1:5000, n = 3) (**D–G**) ELISA quantification of iMφs secreted interleukins (IL-8, TNFa, IL-1β, IL-6) after conditioning with either no stimuli (M0), pro- (M1) or anti-(M2) inflammatory stimuli. Data is normalized on the iMϕ M0 value. (Tukey’s multiple comparison test *p-value < 0.05, ****p-value < 0.0001).

After EB formation from iPSCs, yolk-sac like structures were formed at day 14 in FM medium (Fig. 1A). Subsequently, the production of iMϕ precursor cells initiated (Fig. 1B), with a complete loss of pluripotency markers and surface markers being positive for CD14 and CD45, and negative for CD34, SSEA4 and TRA-1-60 (Supplementary Fig. 1A–D). After iMϕ precursor differentiation in DM, adherent cells were produced (Fig. 1B). These cells expressed CD14, CD45, CD86 and CD163 (Fig. 1C). Cytokine profiling of the supernatant indicated the presence of pro- and anti-inflammatory molecules that were then quantified using ELISA to confirm the presence of macrophage-specific cytokines such as MCP-1, TNF-α, IL-6 and IL-8 (Supplementary Fig. 2). Quantification of the secreted cytokines for iMφs after differentiation is reported in Fig. 2C.

Taken together, iPSCs were successfully differentiated towards iMϕ in the steady (M0) state as confirmed by lack of pluripotency markers (minimal CD34 expression) and presence of macrophage-specific markers (CD14, CD45, CD163 and CD86), as well as IL-8 secretion.

### Capacity of iMφs to polarize to pro- and anti-inflammatory conditions

**Figure 3:**
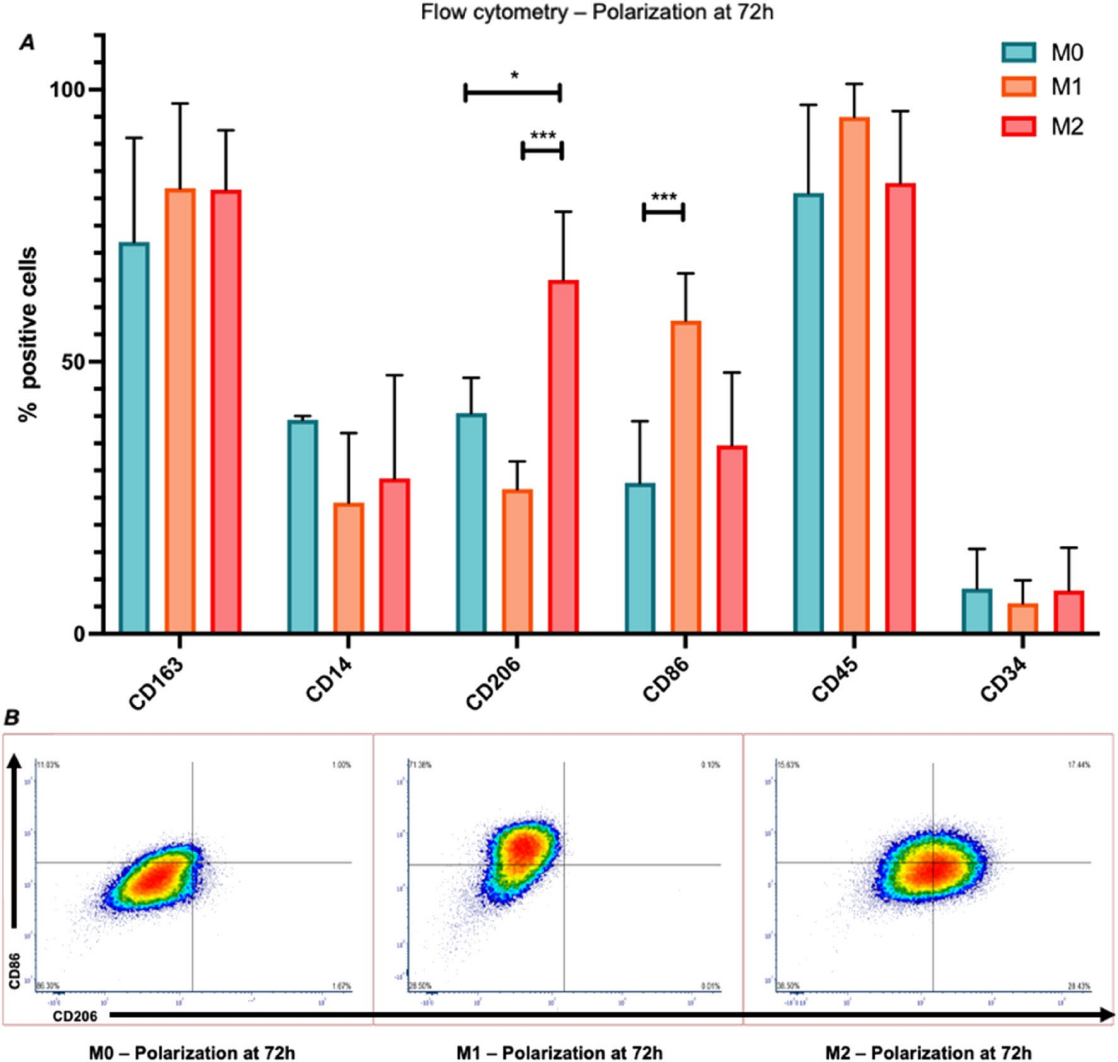
Polarization of iMφs towards pro- and anti-inflammatory states for 72 h. (**A**) Flow cytometry analysis of surface markers to assess the polarization of iMφs after M1 or M2 stimulation for 72 h, in comparison to non-polarized M0 cells (n = 3). (**B**) Flow cytometry density plots of non-polarized and polarized iMφs (M1 and M2 conditions) for the markers CD206 (anti-inflammatory) and CD86 (pro-inflammatory), (2way ANOVA Tukey’s multiple comparisons test, *p-value < 0.05, ***p < 0.0005).

In pro-inflammatory (M1) conditions, pro-inflammatory molecules such as IL-1β, IL-6, and TNF-α were detected at both the gene and protein level (Fig. 2A–C), with significantly higher secretion of these proteins when compared to the anti-inflammatory (M2) and non-polarized (M0) conditions (Fig. 2D–G). Flow cytometry analysis of the polarized cells revealed significantly increased expression of CD86, a known pro-inflammatory macrophage marker, compared to the control non-polarized iMφs (Fig. 3A).

On the other hand, during anti-inflammatory conditions (M2), CD163, TGFβ1 and IL-10 were upregulated at the gene level, compared to the initial non-polarized M0 state (Fig. 2B). IL-10 was not detected as a secreted protein in any of the control polarization conditions. However, M2 iMφs still expressed significantly more CD206When compared to the control non-polarized iMφs (Fig. 3B). These findings indicate that these cells can respond to inflammatory cues in vitro, with significant changes in cell surface markers and cytokine release, especially when exposed to pro-inflammatory conditions.

### hTEM manufacturing and characterization

**Figure 4:**
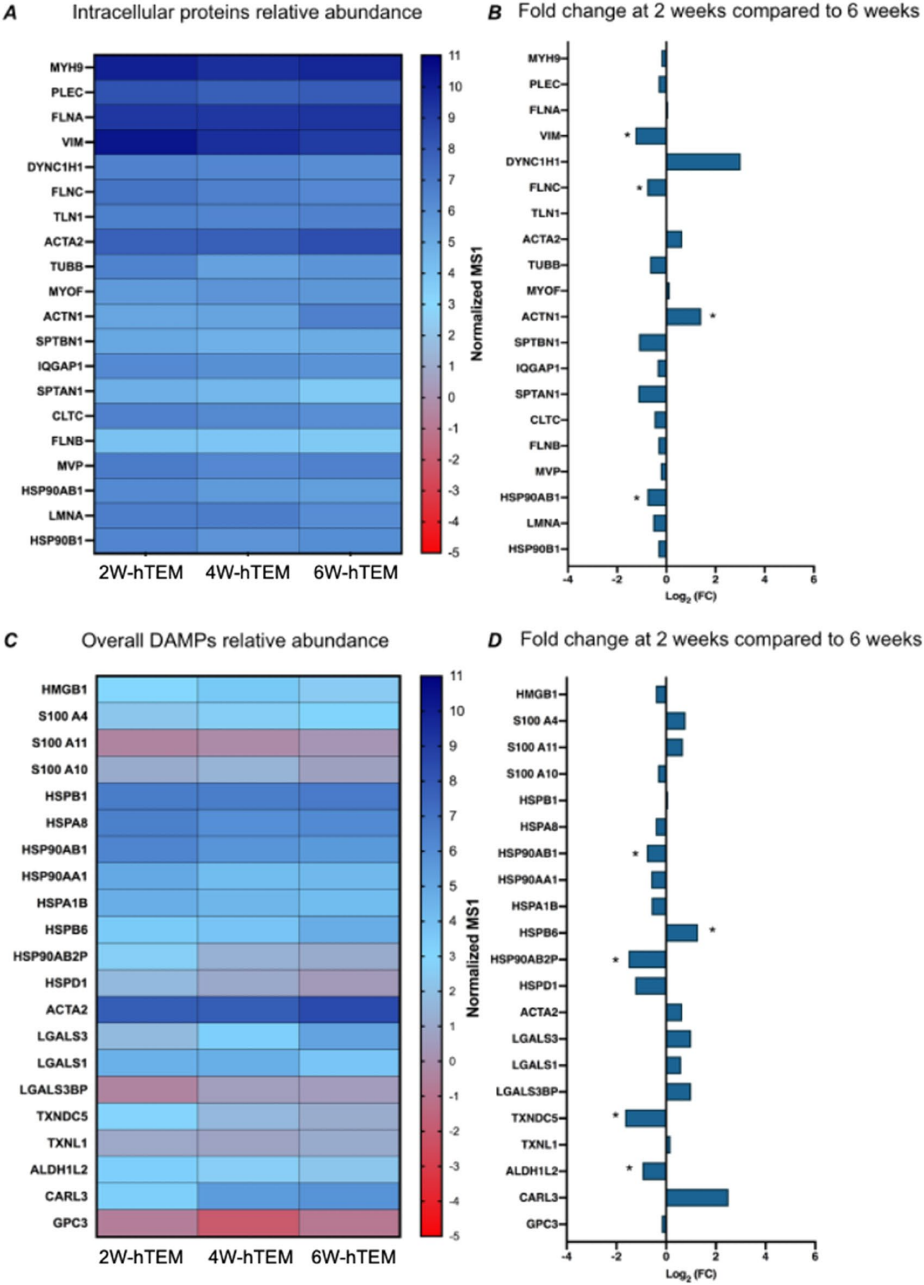
Characterization of potentially immunogenic components within hTEMs. (**A**) Label-free quantification of the 20 most abundant intracellular proteins expressed as normalized MS1 intensities of at 2W, 4W and 6W-hTEMs samples (n = 4/timepoint). (**B**) label-free quantification and fold change of the MS1 values between the initial 2W and final 6W-hTEM culture time points. (**C**) Label-free quantification of DAMPs identified in the matrices, expressed as normalized MS1 intensities at 2W, 4W and 6W-hTEM samples (n = 4/timepoint). (**D**) label-free quantification and fold change of the MS1 values between the initial 2W and final 6W-hTEM production time points (n = 4/timepoint, t-test with pooled variants, *adj. p-value < 0.05).

hTEMs were produced using rotation-based tissue culture (RTC)^9^ and subsequently decellularized as previously described^9^. Efficiency of decellularization of the different hTEM samples was confirmed by using dsDNA levels being below the recommended threshold of 50 ng/mL of dry tissue (undetectable amounts at 2W, 17.9 ± 14.5 ng/mg at 4W, and 24.48 ± 20.1 ng/mg at 6W cultured hTEMs). An in-depth analysis using MS was performed to assess the presence of potentially immunogenic proteins in the hTEM (Fig. 4), which revealed the presence of abundant intracellular proteins (Fig. 4A). Label-free quantification of the 20 most abundant intracellular proteins showed that protein abundance changed overtime with the most significant fold changes in the comparison between 2 and 6W culture time points (Fig. 4B, Supplementary Figs. 3, 4). Actinin alpha 1 (ACTN1) abundance significantly increased overtime, whereas vimentin (VIM) and filamin C (FLNC) decreased. In addition, several of the identified intracellular proteins may be considered as potential immunogens (Supplementary Table 1, Supplementary Fig. 4), such as the intracellular damage associated molecular pattern (DAMP, Fig. 4C) proteins. Label-free quantification of DAMPs (Fig. 4D, Supplementary Fig. 3) showed ACTA2 and heat shock protein family B (small) member 6 (HSPB6) with increasing abundance when 2W hTEMs are compared to 6W-hTEMs. HSPB6 showed significant increase in the same comparison, whereas heat shock protein 90 alpha family class B member 1 (HSP90AB1), heat shock protein 90 alpha family class B member 2, pseudogene (HSP90AB2P), thioredoxin domain containing 5 (TXDNC5) and aldehyde dehydrogenase 1 family member L2 (ALDH1L2) showed significant decrease between the two time points. Overall, hTEMs from every time point showed a similar content of intracellular, DAMP, and xenogeneic proteins. However, the indicated proteins showed significant changes based on the tissue culture time, and with the most significant changes reported when the 2W hTEMs were compared to 6W-hTEMs samples. This indicates that culture times may change the composition of potentially immunogenic proteins found within the hTEMs.

### iMφs secrete IL-6 and IL-1β when cultured on hTEMs suggesting a pro-inflammatory response

**Figure 5:**
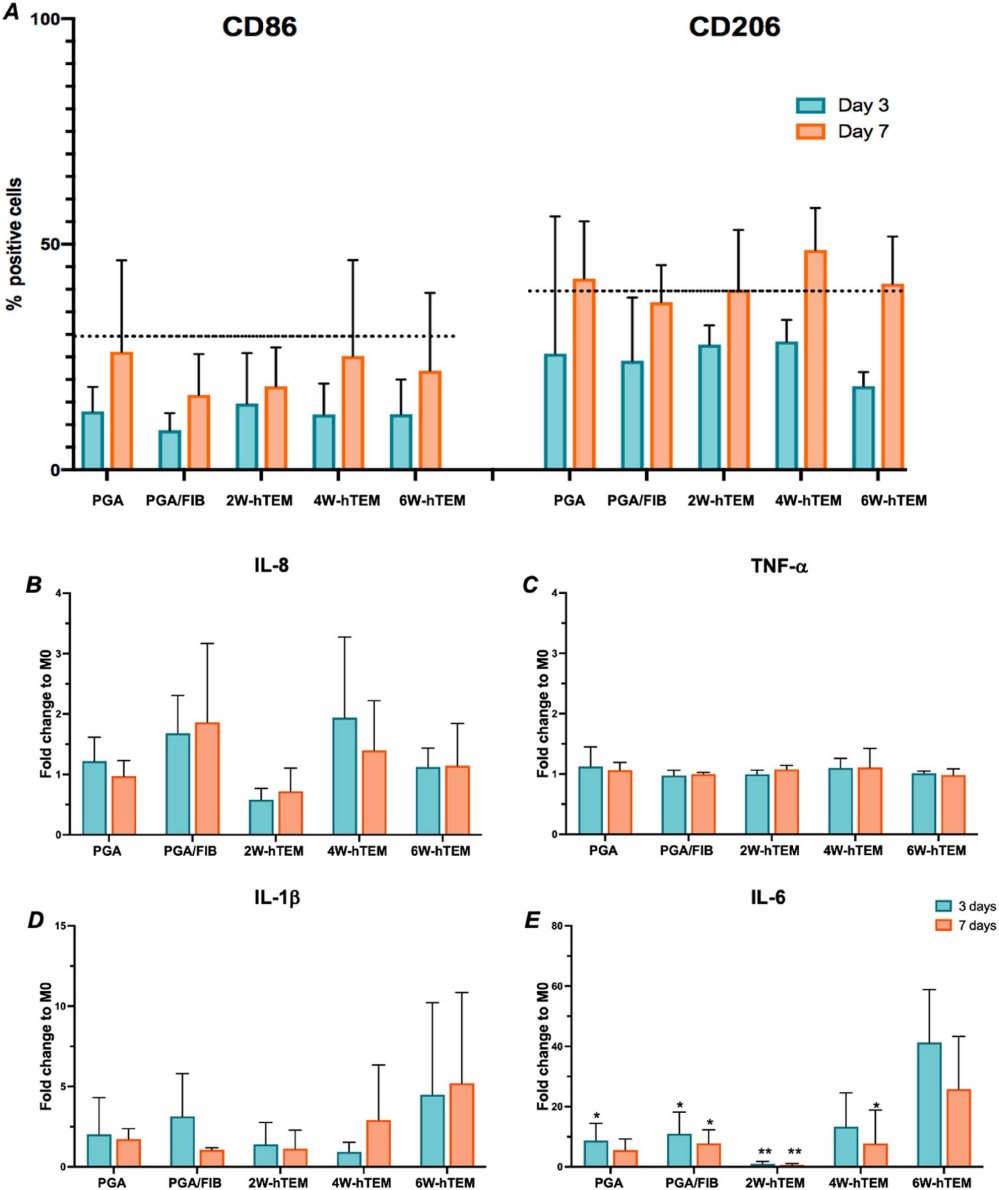
Assessment of hTEM immunogenicity. Scaffold along (PGA/P4HB) and bovine fibrin gel-coated PGA/P4HB (PGA/FIB) controls, and hTEMs produced at 2W, 4W, and 6W (n = 3/timepoint), were cultured with iMφs (M0) for 3 and 7 days (n = 3/timepoint). (**A**) Flow cytometry analysis (n = 3/time point) showed no significant surface marker difference compared to iMφs (M0). (**B–E**) ELISA quantification of secreted cytokines in supernatants (n = 3/timepoint, IL-6 statistics to 6W-hTEM at 3 days. 2way ANOVA Tukey’s multiple comparisons test *p-value < 0.05, **p < 0.005).

To understand whether iMφs can adopt pro- or anti- inflammatory polarization states based on different matrix compositions, we incubated iMφs with the different hTEM samples, using scaffold alone (PGA/P4HB) and fibrin-seeded scaffold (PGA/FIB) as controls, for 3 and 7 days. Independent of the time cells were in culture with the hTEM, flow cytometry showed no difference in the expression of polarization-specific surface markers such as CD86 and CD206 between cells cultured on hTEM samples nor those on the control materials (Fig. 5A). ELISA analysis of the supernatant for pre-selected cytokines (Fig. 2D–G) (IL-8, IL-6, IL-1β, TNF-α and IL-10) indicated no significant difference in the IL-8 and TNF-α secretion levels between control conditions and hTEM/iMφ culture conditions (Fig. 5B,C). However, IL-1β and IL-6 showed an incremental upregulation when iMφs were cultured on hTEMs being produced using 4W or 6W of tissue culture (Fig. 5D,E). Remarkably, IL-6 secretion levels were significantly higher in the 3-day culture when compared to any other experimental condition or time point (Fig. 5E). The identified cytokines were not found in the mass spectrometry analysis of the hTEMs. Overall, these findings indicate that iMϕ are sensitive to the changes in the hTEM substrate and can induce distinct secretion of pro-inflammatory IL-1β and IL-6, similar to the control polarization conditions in (Fig. 2F,G).

## Discussion

The cues that elicit the macrophage pro- and anti-inflammatory responses upon implantation of foreign materials are yet not fully understood^16,18,21,36^. TRMs, which have a different origin from peripheral blood-derived monocytes, have been proposed to play a role in maintaining tissue homeostasis^22,25,27,38^. TRMs, so far, have been underrepresented in the in vitro immunocompatibility platforms for tissue engineering approaches due to limitations in human donor material. However, iMφs have been proposed to model TRMs in vitro^30–32^. Therefore, we introduced iMφs to assess in vitro immunocompatibility, using hTEMs as the material of choice. We further analyzed the proteomic composition of the hTEMs, with focus on the intracellular and xenogeneic proteins to investigate the effects of hTEM composition on the polarization of iMφs in vitro. In this set-up, we demonstrated that the pro-inflammatory cytokine, IL-6, was significantly more abundant in hTEM samples that were produced with a 6W tissue culture time. This suggests that iMφs, TRM-like cells, are a sensitive cell source that can polarize in vitro and can be used as a suitable platform to assess the immunogenic capacity of hTEMs.

### iMφs as a potential tissue specific immunocompatibility assessment platform

TEHVs have shown promising pre-clinical results that could potentially support the introduction of hTEM-based TEHVs in the clinics in the future^7,8,36,39–42^. However, due to the lack of specific guidelines for clinical immunocompatibility requirements of TEHVs, it is imperative to assess the immunocompatibility in vitro in a standardized manner. Currently, the only known immunogenic parameter is the dsDNA content, that should be below the threshold of 50 ng/mg^43^. In this study, we used iMφs, to assess immunocompatibility of hTEMs from the TRM perspective. TRM-like cells have been derived in vitro and are represented by iMφs^30–32,44^. Furthermore, large scale production capacity, and consistent polarization into pro- and anti-inflammatory states has been achieved^31,45^. Moreover, iMφs have been differentiated for tissue specific macrophage investigation purposes, such as microglia, dendritic cells^32^ and alveolar macrophages^46^. In the cardiovascular field, cardiac-specific TRMs are being investigated for their role in disease development^47^, but their involvement in cardiac maintenance has been overlooked so far. A complete characterization of TRMs is still eagerly awaited, especially in order to use TRMs as an in vitro model to study tissue repair. Therefore, in our approach, the use of iMφs serves as a first step towards the investigation of the role of TRM-like cells in tissue repair and TE remodeling in vitro. Our results demonstrate that this platform can demonstrate pro-inflammatory responses of TRM-like cells. In future studies, iMφs could be further adapted to introduce high sensitivity pro-inflammatory read-outs and contribute to the clinical translation of TE constructs.

### iMφs polarization states can be used to identify tissue engineered matrix pro-inflammatory immunogenicity in vitro

In the past, iMφs have been derived from c-Myb independent hematopoiesis through the production of yolk-sac like structures^30^. They have been proposed to represent subsets of the macrophages that are residing in tissues with embryonic origins of the yolk-sac^12,30^. After polarization, the resting (M0) iMφs showed similar secretion of cytokines with the anti-inflammatory polarization (M2) conditions. The reported polarization is in alignment with peripheral blood-derived macrophages from literature^12,31,45,48^. Macrophage polarization states are crucial for ECM-based hTEM remodeling in vivo^49^. Even though the polarization response agrees with literature in the up-regulation of general polarization markers (e.g., IL-6, IL-1β, TNF-α, IL-8 and MCP-1)^45^, it has not been employed for a functional evaluation of immunocompatibility. In this study, iMφs polarization capacity was explored to provide markers for sensitive identification of pro-inflammation polarization. Quantification with ELISA cytokine profiling showed distinct amounts between pro- and anti-inflammatory conditions that led to the selection of IL-6 and IL-1β as the highest secreted interleukins from the ones tested. In the same conditions, IL-6 showed the greatest sensitivity. Moreover, IL-6 proved to have low levels of secretion in the PGA-P4HB controls, which then increased in the 2W-hTEMs, 4W-hTEMs and 6W-hTEMs, respectively. This suggests that the IL-6 could potentially be a key marker of the gradual pro-inflammatory state adaptation of iMφs. In contrast to this, no significant differences in IL-8 between the M0 and the polarized conditions were observed. However, from our initial findings, IL-8 showed consistency as an anti-inflammatory marker in the M0 and M2 polarization states. These findings suggest that IL-8 may not be as sensitive of a marker for anti-inflammatory polarization as CD206, further investigation is required to identify other secreted anti-inflammatory cytokines that can to be used for this purpose. IL-10 has been proposed as a potent anti-inflammatory marker in blood-derived macrophages^50^. However, IL-10 was below the detection level of both the detection assays performed (data not shown), consistent with another study^45^. Thus, these findings indicate that iMφs demonstrate robust polarization and distinct characteristics from blood-derived macrophages in polarization states.

### hTEM composition is crucial for iMφs polarization

In general, it has been recently reviewed that ECM composition has indeed the potential to affect the initial macrophage infiltration and polarization^49^. Our hTEM proteomic composition has been extensively analyzed in multiscale characterization study^9^. The ECM composition of the hTEMs consists of a multitude of proteins and during production the composition changes between 2W, 4W and 6W. The compositions changed in structural constituents such as collagens and other ECM-related proteins that provide mechanical integrity to the hTEMs used for TEHV manufacturing. These changes suggested an increasing ECM deposition, stiffness, and maturation in the hTEMs with longer tissue production times^9^. Importantly, these characteristics could also have an effect on the polarization of iMφs, cultured with hTEMs.

In the hTEMs/iMφ cultures, IL-6 and IL-1β secretion trends were incremental between 2W, 4W, and 6W-hTEMs in both 3- and 7-day cultures. IL-6 and IL-1β share the same activation pathways as toll-like receptors (TLRs) and indicate the timely resolution of the pro-inflammatory response, as IL-6 mediates the transition to non-inflammatory (M2) macrophage polarization^51–53^. The increasing trend of these two interleukins could be related to the different ECM composition of the 2W, 4W, and 6W-hTEMs. In contrast, cells cultured on the controls (PGA and PGA/FIB), which do not contain any ECM proteins, showed significantly less secretion of these interleukins. These results may potentially indicate that IL-6 and IL-1β secretion could be affected by the ECM composition^9,49^. After production, the decellularization of the hTEMs is a very important step done to avoid allogenic immunogenicity of the host. However, after processing, the existence of intracellular proteins could indicate that decellularization efficiency should be assessed not only by the amount of dsDNA but also from the existence of other potential immunogenic proteins. Future immunocompatibility studies could provide more information regarding the tolerable amounts of immunogenic proteins and DAMPs, in order to ensure safe implantation and clinical translation of hTEMs. The current study acts as a first step towards the standardization of hTEM immunogenic characterization with clinical translation in mind. Here, we show that label-free quantification revealed the abundance of ACTA2, ACTN1^54^ and HSPB6^55^, three potentially immunogenic proteins classified as DAMPs. They showed greater abundance at 6W compared to 2W cultured hTEMs, further confirming that hTEM composition changes during tissue culture time, in conjunction to ECM protein content. Higher abundance of these intracellular proteins could potentially result in the IL-6 and IL-1β increased expression in the hTEMs/iMφs cultures. Additionally, our analysis revealed high relative abundances of xenogeneic proteins that originate from the medium supplementation of bovine proteins during hTEM production, where fetal bovine serum was used for culture and fibrin was used as a cell carrier in the PGA-P4HB scaffolds in the initial point of the cultures. Accordingly, iMϕ secretion of IL-6 and IL-1β could be also a result of the xenogeneic protein immunogenicity factor, however, here it is only assessed which is the cumulative effect of the proteomic composition as it would be a more realistic scenario. Moreover, adjusting the culture conditions to xenofree conditions might be beneficial to adjust the proteomic composition during hTEM development^56^. Furthermore, the quantification of residual DNA on the hTEMs has been performed to prove decellularization efficiency as mentioned in the multiscale characterization^9^. The absence of cell nuclei as well as quantification has confirmed that dsDNA levels are below the proposed threshold for immunogenicity (50 ng/mg of dry tissue weight)^43^. Thus, dsDNA content should not be considered as a contributing factor of iMϕ pro-inflammatory interleukin secretion. These findings underline the necessity for further characterization of hTEMs for potentially immunogenic proteomic content beyond the dsDNA quantification. Lastly, it becomes apparent that the importance of hTEM characterization through standardized methods and platforms, is paramount to ensure safe clinical translation.

### Limitations

The hTEM/iMφ cultures have been performed in a static culture set up which does not completely reflect the in vivo dynamic conditions. To this end, the effect physiological-like conditions such as periodic stretch and pulsatile flow, as well as other relevant mechanical cues, should be further investigated in future studies. hDFBs were used as the cell source for hTEM production and the stimulation conditions include TGFβ1, which promotes ECM production through hDFB activation. Therefore, the relevance of the cell source and the produced ECM needs to be further evaluated in future studies. Moreover, the polarization capacity of iMφs in contact with hTEM is investigated, however the capacity of these cells to adopt tissue specific phenotypes still remains to be elucidated. Another, main limitation is the lack of in vivo data to validate the iMφs suitability as an TRM-like cell. Future studies using in vivo evaluation of implanted hTEMs as TEHVs would elucidate the relevance of TRMs in the remodeling of TEHVs are needed.

## Conclusions

We have developed an iPSC-derived TRM-like platform that enables advanced immunocompatibility assessment of tissue engineered matrices in vitro. We demonstrated that iMφs have the capacity to polarize in vitro and respond to differentially composed hTEMs. These results represent a valuable tool to assess immunocompatibility of other TE constructs and ultimately contribute to standardization measures for safe clinical translation of hTEMs.

### Supplementary Information

Supplementary Figures.

Supplementary Information.

Supplementary Tables.

## Data Availability

Raw proteomics data were generated at Functional Genomics Center Zurich. Derived data supporting the findings of this study are available from the corresponding author M.Y.E. on request.

## Abbreviations

hTEM: Human tissue engineered matrix
hDFBs: Human dermal fibroblasts
TEHV: Tissue engineered heart valve
ECM: Extracellular matrix
LC: Liquid chromatography
MS: Mass spectrometry
dsDNA: Double stranded DNA
PGA: Polyglycolic Acid
P4HB: Poly-4-hydroxybutyrate
RTC: Rotation-based tissue culture
TGFβ1: Transforming growth factor-β1
TRM: Tissue-resident macrophage
iPSCs: Induced pluripotent stem cells
iMφs: IPSC-derived macrophages
