# Supplementary material for: Development of an iPSC-derived tissue-resident macrophage-based platform for the in vitro immunocompatibility assessment of human tissue engineered matrices
Source: Sci Rep. 2024 May 28;14:12171. doi: 10.1038/s41598-024-62745-1 (PMC11133401; doi:10.1038/s41598-024-62745-1)
Supplement: Supplementary file 1 — Supplementary Figures. [file 41598_2024_62745_MOESM1_ESM.docx]

**Supplementary figures**

*
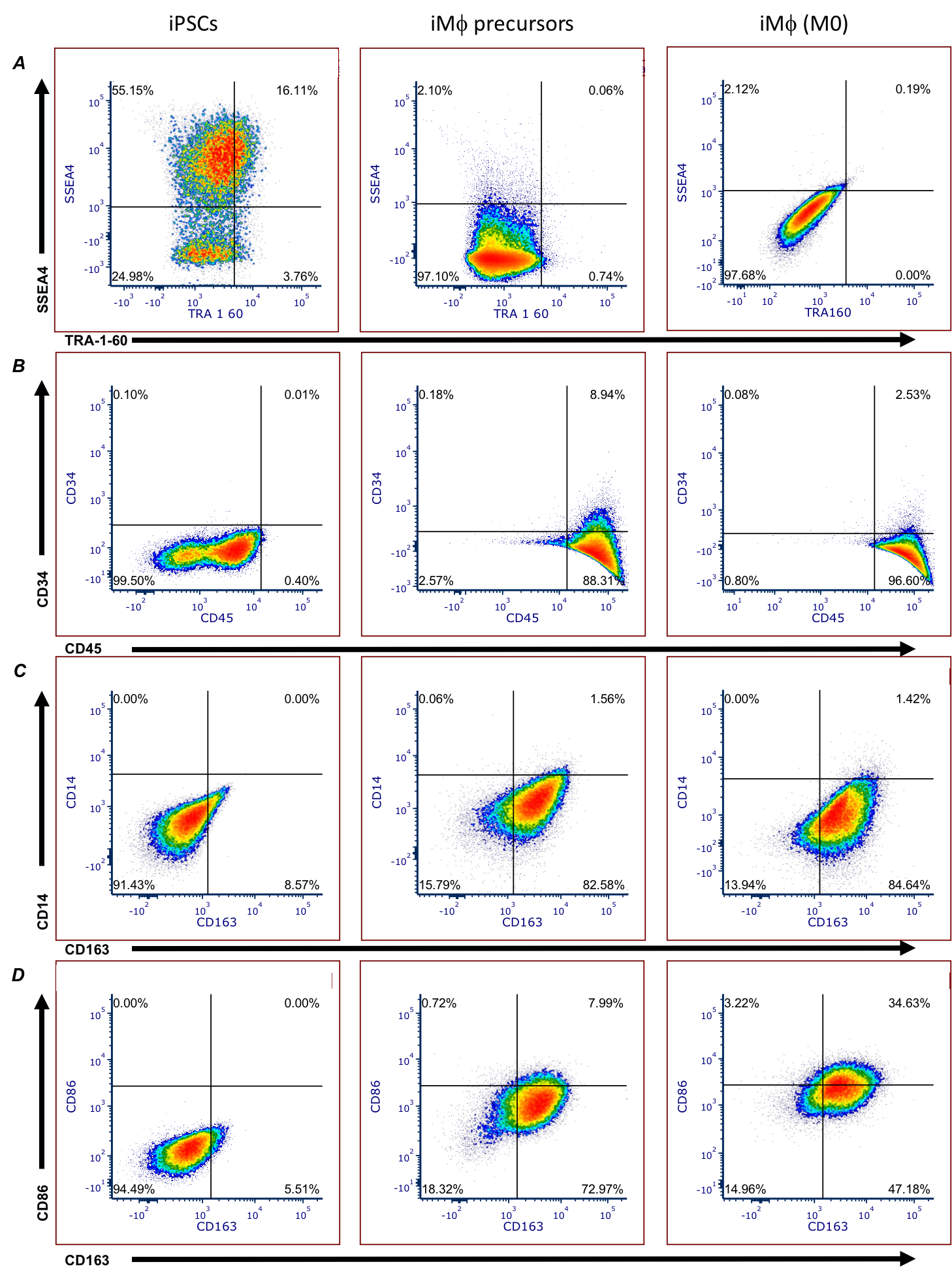
*

***Supplementary Figure 1: Flow cytometry characterization of the differentiation of iPSC-derived macrophages.*** *Three stages are iPSCs, iMφ precursors and iMφs. A) Loss of pluripotency markers. B) CD45 and CD34 expression shift during differentiation, C) CD14 and CD163 expression shift during differentiation and D) CD86 and CD163 expression shift during differentiation. (Representative plots of n=3 technical replicates of one differentiation, 50000 events).*

*
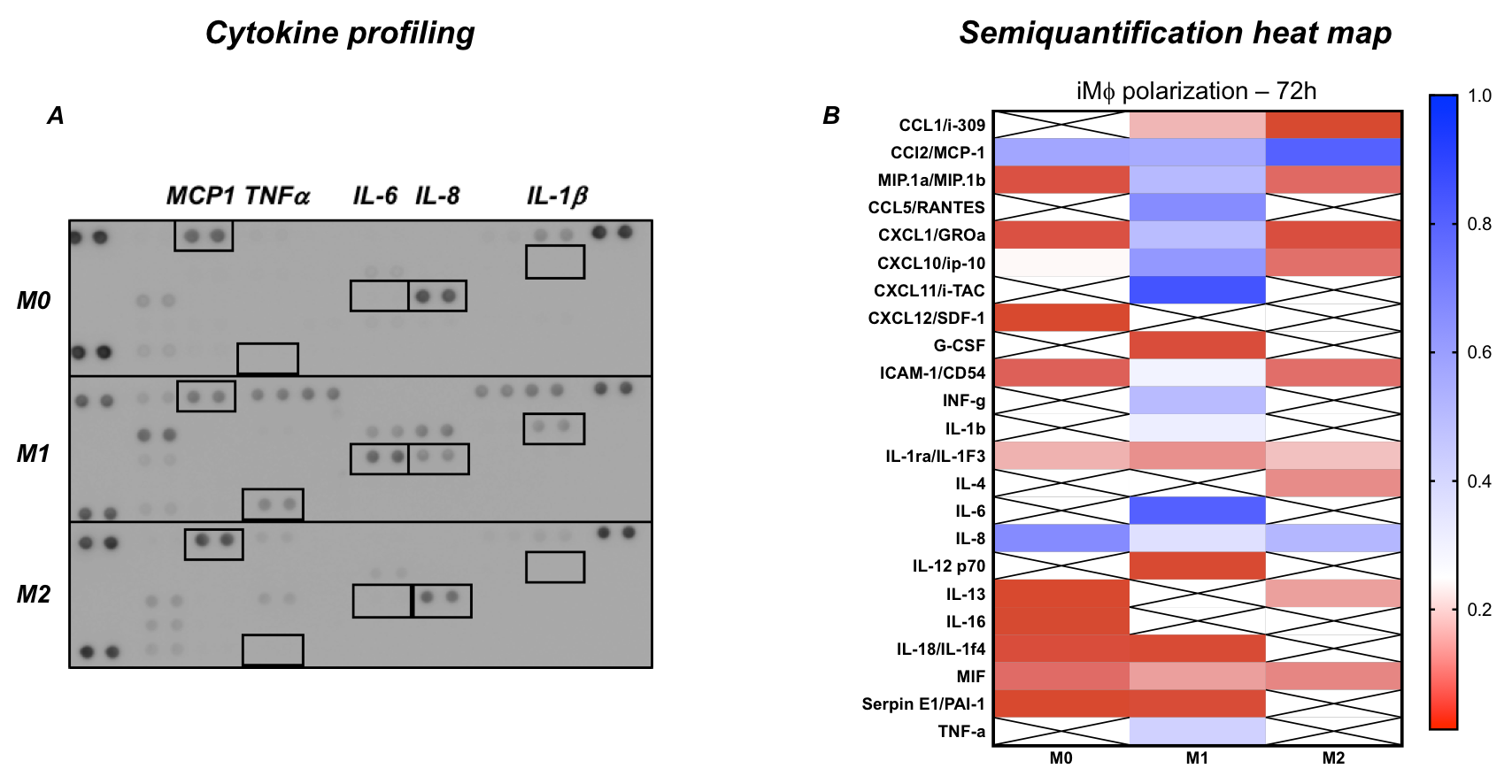
*

***Supplementary Figure 2: Cytokine profiling of iMφ polarization****. A) images of the Cytokine profiling membranes. B) Semi-quantitative analysis of the images using dot density quantification.*

*
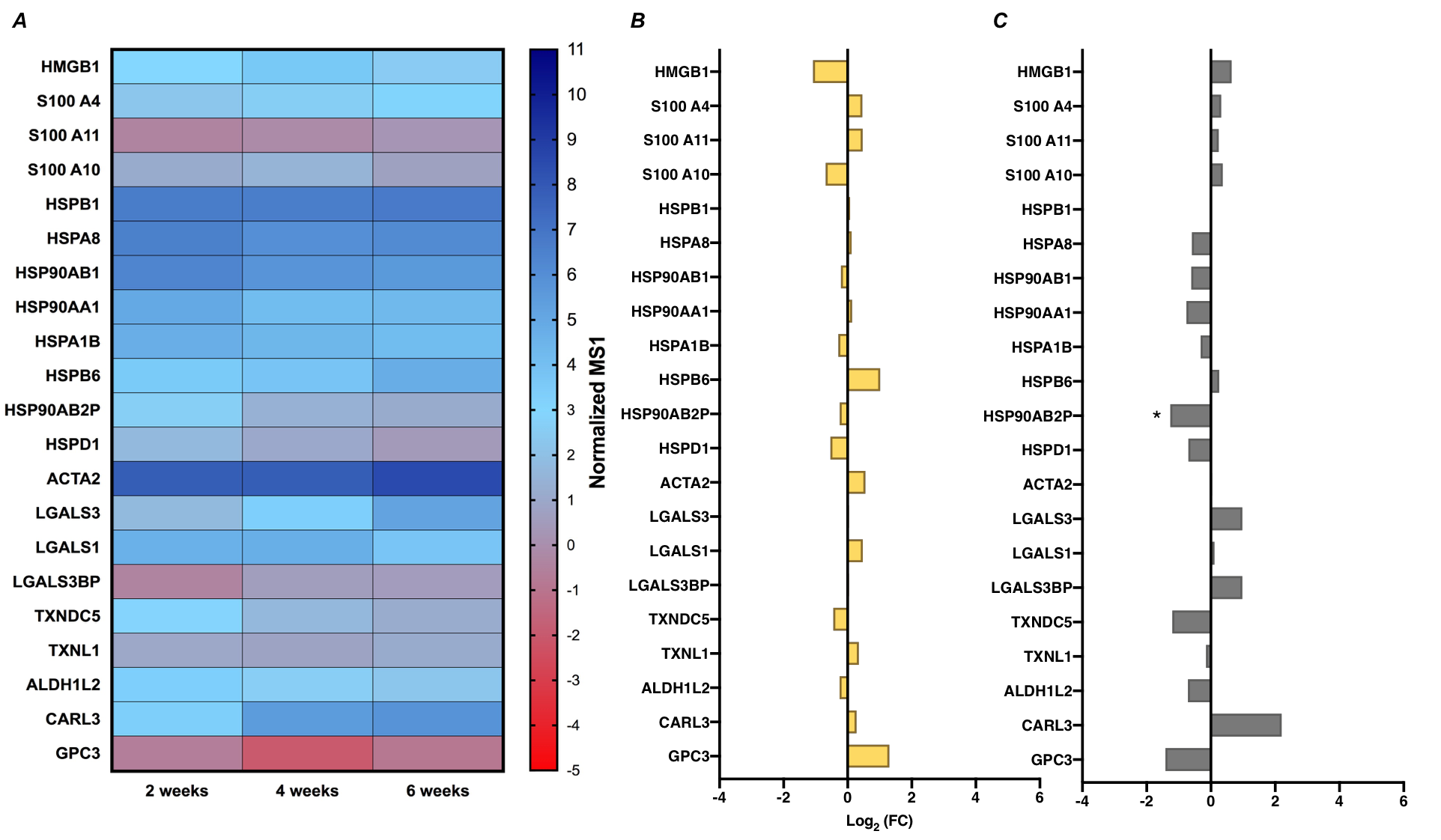
*

***Supplementary Figure 3:*** *A) Mass spectrometry analysis of the hTEMs here is shown the relative abundancy of the 20 most abundant intracellular DAMPs expressed as normalized MS1 intensities at 2, 4 and 6 weeks hTEMs samples. B) label free quantification and fold change of the MS1 values between the initial 4 weeks and final 6 weeks hTEM production time points. C) label free quantification and fold change of the MS1 values between the initial 2 weeks and final 4 weeks hTEM production time points (n=4 / timepoint, t-test with pooled variants, *adj. p-value<0,05).*

*
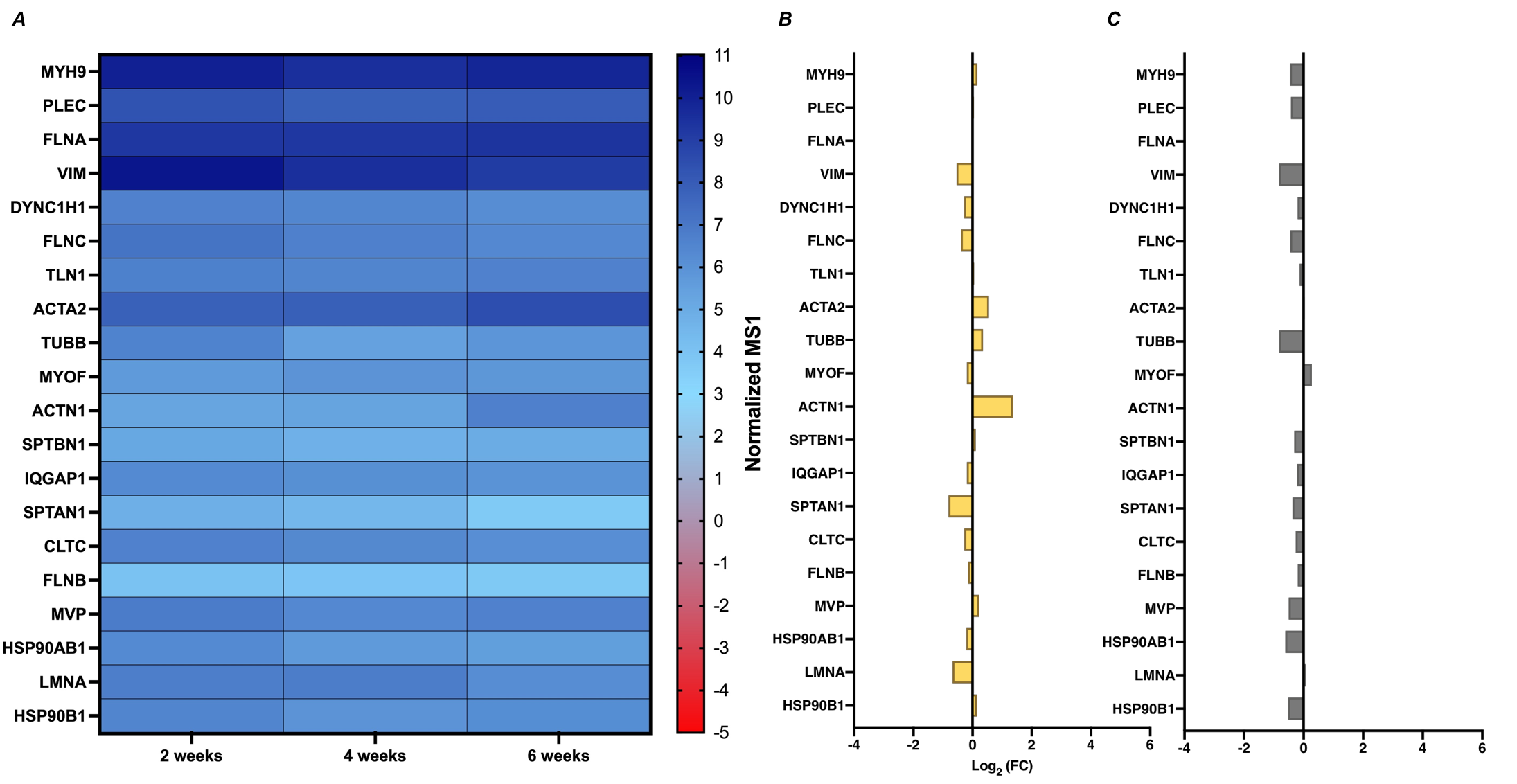
*

***Supplementary Figure 4:*** *A) Mass spectrometry analysis of the hTEMs here is shown the relative abundancy of the 20 most abundant intracellular proteins expressed as normalized MS1 intensities at 2, 4 and 6 weeks hTEMs samples. B) label free quantification and fold change of the MS1 values between the initial 4 weeks and final 6 weeks hTEM production time points. C) label free quantification and fold change of the MS1 values between the initial 2 weeks and final 4 weeks hTEM production time points (n=4 / timepoint, t-test with pooled variants).*

*
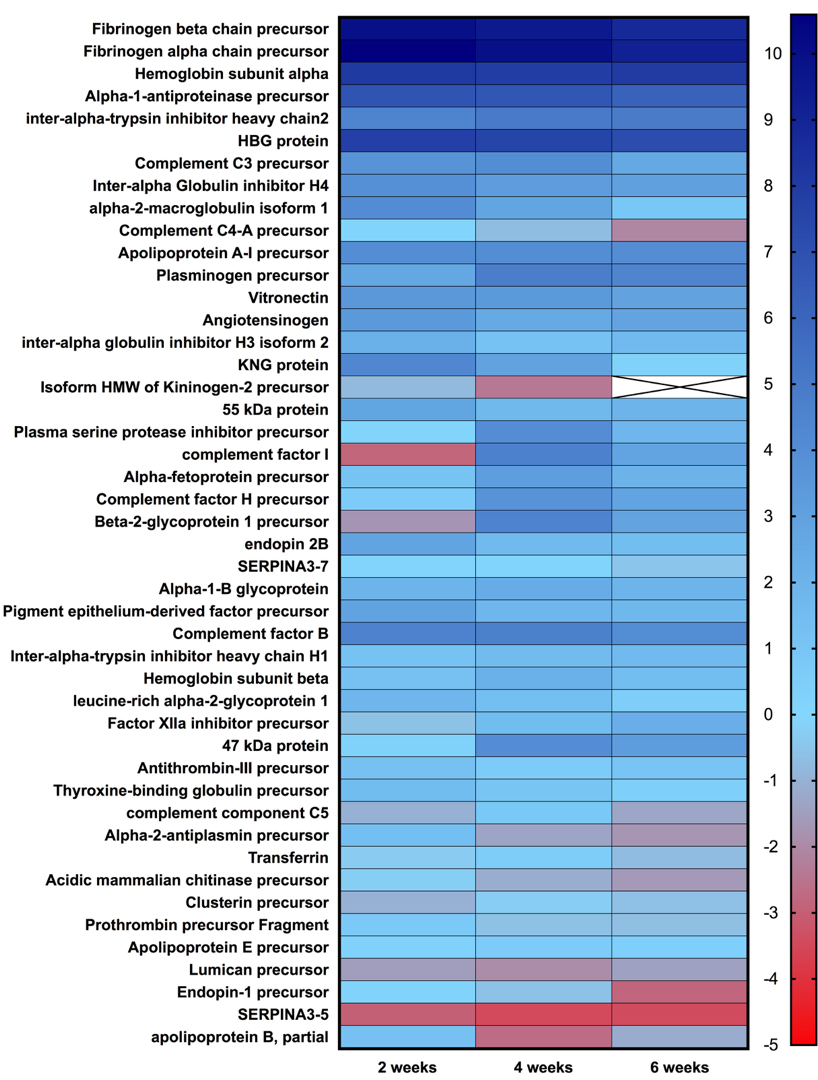
*

***Supplementary Figure 5:*** *A) Mass spectrometry analysis of the hTEMs here is shown the relative abundancy of the 20 most abundant xenogenic proteins expressed as normalized MS1 intensities at 2, 4 and 6 weeks hTEMs samples (n=4 / timepoint).*
