# Supplementary material for: Development of an iPSC-derived tissue-resident macrophage-based platform for the in vitro immunocompatibility assessment of human tissue engineered matrices
Source: Sci Rep. 2024 May 28;14:12171. doi: 10.1038/s41598-024-62745-1 (PMC11133401; doi:10.1038/s41598-024-62745-1)
Supplement: Supplementary file 2 — Supplementary Information. [file 41598_2024_62745_MOESM2_ESM.docx]

### Supplementary methods

#### Scaffold preparation

hTEMs were produced as previously described by using non-woven polyglycolic acid (PGA) meshes (thickness 1 mm; specific gravity 70 mg/cm3; Confluent) coated with 1% poly-4-hydroxybutyrate (P4HB, MW 1x10^6^; TEPHA Inc.) in tetrahydrofuran (Sigma-Aldrich) as starting matrix ^1,2^.

The PGA/P4HB mesh was cut into scaffolds (width:100 mm; hight: 45 mm) and then sutured on a cylindrical scaffold holder (30mm in height, 28 mm inner diameter) based on a nitinol stent, as previously described ^1^ (Figure 1C). The nitinol stent is wrapped with a layer of perforated parafilm in order to avoid stent embedding in the formed matrix. Afterwards, the scaffold is combined to the RTC insert (Figure 1D) and the full constructs is sterilized (30 min incubation in 70% ethanol followed by 30 min incubation in PBS supplemented with 10% penicillin-streptomycin (Sigma) and 1% antibiotic-antimicotic solution (Sigma). Finally, the scaffolds were incubated overnight in *cell culture medium* (Advanced DMEM (Gibco), supplemented with 10% fetal bovine serum (FBS, Gibco), 1% GlutaMax (Gibco), and 1% penicillin-streptomycin (Sigma).

#### 1.2 Cell expansion and seeding for hTEM production

Human dermal fibroblasts (hDFBs, CellSystems Biotechnologie GmbH, passages 7–10) were plated in roller bottles (850 cm^2^, TufRol EZ, Nunc) and expanded in cell culture medium till confluency using the CELLROLL system. Upon harvesting, cells were seeded onto the scaffolds (1 x 10^6^ cells/cm2) using fibrin as a cell carrier covering completely the scaffold, as previously established ^3^. After seeding, the constructs were incubated statically in cell culture medium overnight to favour cell adhesion.

#### 1.3 hTEM culture

The day after the seeding, the constructs were transferred to a pleated roller-bottle (1450 cm^2^, TufRol, Nunc). Constructs were cultured using 150 ml of tissue culture medium (cell culture medium supplemented with l-ascorbic acid 2-phosphate (0.26 mg/ml; Sigma-Aldrich) from day 1, and 5 ng/ml TGF-β1 (Peprotech), starting at day 7), that was replaced twice a week. Tissue culture occurred using a rotation speed of 1.5 rpm in standard incubator settings (37°C, 5% CO_2_, and 95% relative humidity). After 2, 4, or 6 weeks of tissue culture, hTEM tubes were washed in PBS and decellularized ^4^ using an optimized protocol based on the work of Lintas et al ^1^ (Supplementary Materials 1.1).

*1.4 Decellularization*

hTEM were incubated twice overnight in a detergent solution (0.25% Triton X-100, sodium deoxycholate and 0.02% ethylenediaminetetraacetic acid in DPBS) in the roller bottle. Additionally, four Benzonase (Novagen) incubation steps with decreasing concentration (100, 80, 40 and 20 U/ml in 50 mM TRIS–HCl buffer solution with pH 8.0) were used to degrade the remaining DNA remnants. After rinsing, hTEM tubes were stored in PBS supplemented with 1% penicillin / streptomycin (Sigma) at 4°C until further use.

*1.5 Detailed Mass spectrometry workflow*

*Sample digestion – PreOmics*

Samples were processed using a commercial iST Kit (PreOmics, Germany). To each sample, 50 µl of ‘Lyse’ buffer was added, boiled at 95°C for 10 minutes, transferred to the cartridge and digested by adding 50 µl of the ‘Digest’ solution. After 120 minutes of incubation at 37 °C, the digestion was stopped with 100 µl of ‘Stop’ solution. The solutions in the cartridge were removed by centrifugation at 3800 g, while the peptides were retained by the iST-filter. Finally, the peptides were washed, eluted, dried and re-solubilized in 20 µl of MS sample solvent (3% acetonitrile, 0.1% formic acid).

The peptide absorbance (A280) was measured using a DeNovix DS-11 Series Spectrophotometer and the samples were normalized to an absorption of 1.2.

*LC-MS/MS – Lumos OT/IT DDA*

LC-MS/MS analysis was performed on an Orbitrap Fusion Lumos (Thermo Scientific) equipped with a Digital PicoView source (New Objective) and coupled to an M-Class UPLC (Waters). Solvent composition of the two channels was 0.1% formic acid for channel A and 99.9% acetonitrile in 0.1% formic acid for channel B. Column temperature was 50°C. For each sample 2 μl of peptides were loaded on a commercial ACQUITY UPLC M-Class Symmetry C18 Trap Column (100Å, 5 µm, 180 µm x 20 mm, Waters) connected to a ACQUITY UPLC M-Class HSS T3 Column (100Å, 1.8 µm, 75 µm X 250 mm, Waters). The peptides were eluted at a flow rate of 300 nL/min. After a 3 min initial hold at 5% B, a gradient from 5 to 22 % B in 80 min and 22 to 32% B in additional 10 min was applied. The column was cleaned after the run by increasing to 95 % B and holding 95 % B for 10 min prior to re-establishing loading condition.

Samples were measured in randomized order. The mass spectrometer was operated in data-dependent mode (DDA) with a maximum cycle time of 3 s, with spray voltage set to 2.3 kV, funnel RF level at 40 %, heated capillary temperature at 275 °C, and Advanced Peak Determination (APD) on. Full-scan MS spectra (300−1’500 m/z) were acquired at a resolution of 120’000 at 200 m/z after accumulation to an automated gain control (AGC) target value of 500’000 or for a maximum injection time of 40 ms. Precursors with an intensity above 5’000 were selected for MS/MS. Ions were isolated using a quadrupole mass filter with 0.8 m/z isolation window and fragmented by higher-energy collisional dissociation (HCD) using a normalized collision energy of 35 %. Fragments were detected in the linear ion trap with the scan rate set to rapid, the automatic gain control set to 10’000 ions, and the maximum injection time set to 50 ms. Charge state screening was enabled, and singly, unassigned charge states and charge states higher than seven were excluded. Precursor masses previously selected for MS/MS measurement were excluded from further selection for 20 s, applying a mass tolerance of 10 ppm. The samples were acquired using internal lock mass calibration on m/z 371.1012 and 445.1200.

The mass spectrometry proteomics data were handled using the local laboratory information management system (LIMS) ^5^ and all relevant data have been deposited to the ProteomeXchange Consortium via the PRIDE (http://www.ebi.ac.uk/pride) partner repository with the data set identifier PXDXXXX.

*Peptide identification and quantification – MaxQuant lfq*

The acquired raw MS data were processed by MaxQuant (version 1.6.2.3), followed by protein identification using the integrated Andromeda search engine ^6^. Spectra were searched against the Uniprot Homo sapiens reference proteome (taxonomy 9606, canonical version from 2019-07-09), concatenated to its reversed decoyed fasta database and common protein contaminants. Carbamidomethylation of cysteine was set as fixed modification, while methionine oxidation, Deamidation of glutamine and asparagine and N-terminal protein acetylation were set as variable. Enzyme specificity was set to trypsin/P allowing a minimal peptide length of 7 amino acids and a maximum of two missed-cleavages. MaxQuant Iontrap default search settings were used. The maximum false discovery rate (FDR) was set to 0.01 for peptides and 0.05 for proteins. Label free quantification was enabled and a 2 minutes window for match between runs was applied. In the MaxQuant experimental design template, each file is kept separate in the experimental design to obtain individual quantitative values. Protein fold changes were computed based on Intensity values reported in the proteinGroups.txt file. A set of functions implemented in the R package SRMService [BB - W. Wolski, J. Grossmann, C. Panse. 2018. SRMService - R-Package to Report Quantitative Mass Spectrometry Data. http://github.com/protViz/SRMService] was used to filter for proteins with 2 or more peptides allowing for a maximum of 4 missing values, and to normalize the data with a modified robust z-score transformation and to compute p-values using the t-test with pooled variance. If all measurements of a protein are missing in one of the conditions, a pseudo fold change was computed replacing the missing group average by the mean of 10% smallest protein intensities in that condition.

*Software URLs*

**QuantStudio™ 7 Flex Real-Time PCR System 384:** https://www.thermofisher.com/order/catalog/product/4485701?SID=srch-srp-4485701

**GraphPad Prism 8**: https://www.graphpad.com/updates/prism-802-release-notes

**BD FACSDiva^TM^ v8**: https://www.bdbiosciences.com/en-us/products/software/instrument-software/bd-facsdiva-software

**FCS 6 Express**: https://denovosoftware.com/full-access/download-landing/previous-version-release-notes/

**TECAN M1000 pro, i-control^TM^:** https://lifesciences.tecan.com/plate_readers/infinite_200_pro?p=Software

1. Lintas, V. *et al.* Development of a Novel Human Cell-Derived Tissue-Engineered Heart Valve for Transcatheter Aortic Valve Replacement: an In Vitro and In Vivo Feasibility Study. *J Cardiovasc Transl Res* **11**, 470–482 (2018).

2. Motta, S. E. *et al.* Human cell-derived tissue-engineered heart valve with integrated Valsalva sinuses: towards native-like transcatheter pulmonary valve replacements. *NPJ Regen Med* **4**, 14 (2019).

3. Mol, A. *et al.* Fibrin as a cell carrier in cardiovascular tissue engineering applications. *Biomaterials* **26**, 3113–3121 (2005).

4. Dijkman, P. E., Driessen-Mol, A., Frese, L., Hoerstrup, S. P. & Baaijens, F. P. T. T. Decellularized homologous tissue-engineered heart valves as off-the-shelf alternatives to xeno- and homografts. *Biomaterials* **33**, 4545–4554 (2012).

5. Türker, C. *et al.* B-Fabric: The Swiss Army Knife for Life Sciences. (2010).

6. Cox, J. & Mann, M. MaxQuant enables high peptide identification rates, individualized p.p.b.-range mass accuracies and proteome-wide protein quantification. *Nat Biotechnol* **26**, (2008).
