# Supplementary material for: Development of an iPSC-derived tissue-resident macrophage-based platform for the in vitro immunocompatibility assessment of human tissue engineered matrices
Source: Sci Rep. 2024 May 28;14:12171. doi: 10.1038/s41598-024-62745-1 (PMC11133401; doi:10.1038/s41598-024-62745-1)
Supplement: Supplementary file 3 — Supplementary Tables. [file 41598_2024_62745_MOESM3_ESM.docx]

**Table of immunogenic proteins and protein families**

***Supplementary Table 1:*** *List of potentially immunogenic proteins, protein families and Damage Associated molecular Patterns (DAMPs) in hTEMs as found from the mass spectrometry.*

| Molecule | Reason of immunogenicity | Reference |
| --- | --- | --- |
| Xenogeneic proteins | | |
| Serum albumin (ALB) | The amino acid sequence 524-598 is an epitope for human antibodies.  Widely studied protein, it has been reported as the major allergens for milk and meat ^1^. | D. Male et al. (1985)^1^ |
| Hemoglobin alpha-chain 1 (HBA1) & Beta (HBB) | ﻿Identified through immunoproteomic to be able to elicit a humoral immune response in rabbits ^2^. | L. Griffiths et al. (2008)^2^ |
| Pro-inflammatory DAMPs | | |
| Intracellular DAMPs | | |
| DNA, chromatin and nucleosomes | DNA fragments have been implicated to cause an inflammatory reaction upon implantation of decellularized tissue. The immune reaction depends on DNA fragment size and the amount of DNA present in decellularized tissue ^3^. DNA is a strong DAMP signal ^4^. | T. Gilbert et al. (2009)^3^  E. Karayel et al. (2009) ^4^  H. Kono et al. (2008)^5^ |
| RNA | DAMP signal when released in the extracellular space ^6^. | D. Tang et al. (2012)^6^ |
| S100 proteins | S100 proteins are intracellular calcium binding proteins. S100 proteins turn into a pro-inflammatory signal when it is released in the extracellular space (DAMP signal) ^7^. | D. Foell et al. (2007) ^7^ |
| Heat shock proteins (HSPs) | Heat shock protein is a DAMP signal ^8^. | 1. Morris et al. (2017)^8^ |
| High mobility box group 1 (HMBG 1) | HMGB1 is a member of the nonhistone, chromatin-associated high mobility  group family of proteins.  It binds to the  minor groove of DNA and facilitates the assembly of site-specific ^9^.  HMBG 1 is a DAMP signal ^10^. | J. O. Thomas et al. (2001) ^9^  G. Srikrishna et al. (2009)^10^ |
| Galectins | Galectins bind to a wide array of glycoproteins and glycolipids both on the cell surface and in extracellular matrices. Galectins deliver signals intracellularly and mediate cell–cell and cell–extracellular matrix adhesion ^11^. DAMP signals ^5^. | H. Kono et al. (2008)^5^  F. Liu et al. (2005) ^11^ |
| Thioredoxin (TXN) | Thioredoxin is a redox enzymes released during inflammation and infection.  It is a chemoattractant for Neutrophils, Monocytes and T cells ^12^.  DAMP signal ^5^. | R. Bertini et al. (1999) ^12^  H. Kono et al. (2008)^5^ |
| Cathelicidins | Chemoattractant for Neutrophils, Monocytes and T cells ^13^.  DAMP signal ^5^. | M. Zanetti (2004) ^13^  H. Kono et al. (2008)^5^ |
| Defensins | Chemoattractant^14^  DAMP signal ^5^. | D. Yang (1999) ^14^  H. Kono et al. (2008)^5^ |
| Mitochondrial DAMPS | | |
| *N-*formylated peptides | Chemoattractant for neutrophils ^15^  DAMP signal ^5^. | H. Carp (1982) ^15^  H. Kono et al. (2008)^5^ |
| Plasma DAMPs | | |
| Complement proteins (C3a, C4a, C5a) | DAMP signal when activated^6^. | D. Tang et al. (2012)^6^ |
| Extracellular DAMPs | | |
| Hyaluronic acid | Hyaluronic fragments act as a DAMP signal^16^. | K. R. Taylor et al. (2004)^16^ |
| Heparan sulphate (HS, HSPG) | Heparan sulphate fragments act as a DAMP signal ^17^. | B. Geoffrey et al. (2002)^17^ |
| Fibrinogen (FGB) | Fibrinogen fragments act as a DAMP signal ^18^. | S. T. Smiley et al. (2001)^18^ |
| Collagen-derived peptides (COL) | Collagen fragments act as a DAMP signal [13], ^19^. | H. Kono et al. (2008)^5^  N. M. Weathington et al. (2001) ^19^ |
| Fibronectin (FN) | Fibronectin fragments act as a DAMP signal [13], ^20^. | H. Kono et al. (2008)^5^  U. Brand et al. (1998) ^20^ |
| Elastin-derived peptides (ELN) | Elastin-derived fragments act as a DAMP signal [13], ^21^. | H. Kono et al. (2008)^5^  A. M. Houghton et al. (2006)^21^ |
| Laminin (LAMA, LAMB, LAMC) | Laminin fragments act as a DAMP signal [13],^22^. | H. Kono et al. (2008)^5^  T. L. Adair-Kirk et al. (2003)^22^ |
|  |  |  |
| Other mechanism | | |
| Peroxiredoxin (PRDX) | Peroxiredoxins are enzymes with an antioxidant function.  Overexpression in non-small cell lung cancer ^23^.  ﻿IgG anti-Peroxiredoxin I antibody is associated with the disease severity of systemic sclerosis ^24^. Autoantibodies to peroxiredoxin I and IV were detected in systemic autoimmune diseases ^25^. | W. Jong et al. (2005)^23^ Y. Iwata et al. (2007)^24^  R. Karasawa et al. (2005)^25^ |
| Lyn tyrosine-kinase (LYN) | ﻿Lyn tyrosine-kinase belongs to the Src family-kinase. Lynn is expressed in B cells and has both positive and negative regulatory roles in B cell receptor - induced signal transduction | Y. Xu et al. (2005)^26^ |
| Alpha-enolase (ENO1) | ﻿Antibodies against alpha-enolase have been detected in a large variety of infectious and autoimmune diseases ^2728^. Exact mechanism unknown. | B. Terrier et al. (2007)^27^  D. Bogdanos et al. (2004)^28^ |
| Triosephosphate Isomerase (TPI) | ﻿Triosephosphate Isomerase is a glycolytic enzyme. Autoantibodies have been found in ﻿Osteoarthritis disease ^29^. | Y. Xiang et al. (2004)^29^ |

***Supplementary Table 2:*** *List of primers used for gene expression*

| Primers | Forward | Reverse |
| --- | --- | --- |
| CD11b | AGTTGCCGAATTGCATCGA | GGCGTTCCCACCAGAGAGA |
| CD68 | CACTGGGGCAGGAGAAACT | TTCACCAGCTGTCCACCTC |
| IL-1β | GTGGCAATGAGGATGACTTGTTCT | TGTAGTGGTGGTCGGAGATTCG |
| IL-6 | ACTCACCTCTTCAGAACGAATTG | GTCGAGGATGTACCGAATTTGT |
| TNF-α | GAGGCCAAGCCCTGGTATG | CGGGCCGATTGATCTCAGC |
| TGF-β1 | GCAACAATTCCTGGCGATACC | GTTCTTCTCCGTGGAGCTGAA |
| CD163 | CACTATGAAGAAGCCAAAATTACCT | AGAGAGAAGTCCGAATCACAGA |
| IL-10 | GACTTTAAGGGTTACCTGGGTTG | TCACATGCGCCTTGATGTCTG |

**Supplementary table 3:** *List of DAMPs and their respective recognition receptors*.

**Extracellular matrix**

| Biglycan | TLR2, TLR4, NLRP3 |
| --- | --- |
| Decorin | TLR2, TLR4 |
| Versican | TLR2, TLR6, CD14 |
| LMW hyaluronan | TLR2, TLR4, NLRP3 |
| Heparan sulfate | TLR4 |
| Fibronectin (EDA domain) | TLR4 |

Intracellular – cytosol

| Uric acid | NLRP3, P2X7 |
| --- | --- |
| S100 proteins | TLR2, TLR4, RAGE |
| Heat shock proteins | TLR2, TLR4, CD91 |
| ATP | P2X7, P2Y2 |
| F-actin | DNGR-1 |
| Cyclophilin A | CD147 |
| Aβ | TLR2, NLRP1, NLRP3, CD36, RAGE |

Intracellular – nuclear

| Histones | TLR2, TLR4 |
| --- | --- |
| HMGB1 | TLR2, TLR4, RAGE |
| HMGN1 | TLR4 |
| IL-1α | IL-1R |
| IL-33 | ST2 |
| SAP130 | Mincle |
| DNA | TLR9, AIM2 |
| RNA | TLR3, TLR7, TLR8, RIG-I, MDA5 |

Intracellular-Mithocondria

| mtDNA | TLR9 |
| --- | --- |
| TFAM | RAGE |
| Formyl peptide | FPR1 |
| mROS | NLRP3 |

Intracellular-endoplasmic reticulum

| Calreticulin | CD91 |
| --- | --- |

Intracellular - granule

| Defensins | TLR4 |
| --- | --- |
| Cathelicidin (LL37) | P2X7, FPR2 |
| EDN | TLR2 |
| Granulysin | TLR4 |

Intracellular – plasma membrane

| Syndecans | TLR4 |
| --- | --- |
| Glypicans | TLR4 |

***Supplementary table 4:*** *Mass spectrometry human intracellular proteins and intracellular DAMPs (MS1, Label free (FC)).*

| **Intracellular proteins** | | **Intracellular DAMPs** | |
| --- | --- | --- | --- |
| MYH9 | Myosin Heavy Chain 9 | HMGB1 | High mobility group box 1 |
| PLEC | Plectin | S100 A4 | S100 Calcium Binding Protein A4 |
| FLNA | Filamin A | S100 A11 | S100 Calcium Binding Protein A11 |
| VIM | Vimentin | S100 A10 | S100 Calcium Binding Protein A10 |
| DYNC1H1 | Dynein Cytoplasmic 1 Heavy Chain1 | HSPB1 | Heat shock protein beta-1 |
| FLNC | Filamin C | HSPA8 | Heat shock protein alpha member 8 |
| TLN1 | Talin 1 | HSP90AB2P | heat shock protein 90 alpha class B member 2 pseudogene |
| ACTA2 | Actin alpha 2 | HSPD1 | Heat Shock Protein D (Hsp60) 1 |
| TUBB | Tubulin beta chain | ACTA2 | Actin alpha 2 |
| MYOF | Myoferlin | LGALS3 | Galectin 3 |
| ACTN1 | Actinin Alpha 1 | LGALS1 | Galectin 1 |
| SPTBN1 | Spectrin Beta, Non-Erythrocytic 1 | LGALS3BP | Galectin-3-binding protein |
| IQGAP1 | IQ Motif Containing GTPase Activating Protein 1 | TXNDC5 | Thioredoxin domain-containing protein 5 |
| SPTAN1 | Spectrin alpha chain, non-erythrocytic 1 | TXNL1 | Thioredoxin-like protein 1 |
| CLTC | Clathrin heavy chain 1 | ALDH1L2 | Aldehyde dehydrogenase 1 member L2 |
| FLNB | Filamin B | CARL3 | Capping Protein Regulator And Myosin 1 Linker 3 |
| MVP | Major vault protein | GPC3 | Glypican 3 |
| HSP90AB1 | Heat shock protein HSP 90-beta |  |  |
| LMNA | Lamin A/C |  |  |
| HSP90B1 | Heat Shock Protein 90 Beta Member1 |  |  |

1. Male, D. K., Champion, B. R., Pryce, G. & Matthews, H. Antigenic determinants of Bovine Serum Albumin. 419–427 (1985).

2. Griffiths, L. G., Choe, L. H., Reardon, K. F., Dow, S. W. & Christopher Orton, E. Immunoproteomic identification of bovine pericardium xenoantigens. *Biomaterials* **29**, 3514–3520 (2008).

3. Gilbert, T. W., Freund, J. M. & Badylak, S. F. Quantification of DNA in Biologic Scaffold Materials. *Journal of Surgical Research* **152**, 135–139 (2009).

4. Karayel, E. *et al.* The TLR-independent DNA recognition pathway in murine macrophages : Ligand features and molecular signature. 1929–1936 (2009) doi:10.1002/eji.200939344.

5. Kono, H. & Rock, K. L. How dying cells alert the immune system to danger. *Nat Rev Immunol* **8**, 279–289 (2008).

6. Tang, D., Kang, R., Coyne, C. B., Zeh, H. J. & Lotze, M. T. PAMPs and DAMPs: Signal 0s that spur autophagy and immunity. *Immunol Rev* **249**, 158–175 (2012).

7. Foell, D., Wittkowski, H., Vogl, T. & Roth, J. S100 proteins expressed in phagocytes: a novel group of damage-associated molecular pattern molecules. *J Leukoc Biol* **81**, 28–37 (2007).

8. Morris, A. H., Stamer, D. K. & Kyriakides, T. R. The host response to naturally-derived extracellular matrix biomaterials. *Semin Immunol* **29**, 72–91 (2017).

9. Thomas, J. O. & Travers, A. A. HMG1 and 2, and related ‘architectural’ DNA-binding proteins. *Trends Biochem Sci* **26**, 167–174 (2001).

10. Srikrishna, G. & Freeze, H. H. Endogenous damage-associated molecular pattern molecules at the crossroads of inflammation and cancer. *Neoplasia* **11**, 615–628 (2009).

11. Liu, F. T. & Rabinovich, G. A. Galectins as modulators of tumour progression. *Nat Rev Cancer* **5**, 29–41 (2005).

12. Bertini, B. R. *et al.* Inflammation , Is a Unique Chemoattractant for Neutrophils , Monocytes, and T Cells. *Journal of Experimental Medicine* **189**, 1783–1789 (1999).

13. Zanetti, M. Cathelicidins, multifunctional peptides of the innate immunity. *J Leukoc Biol* **75**, 39–48 (2004).

14. Yang, D. *et al.* B-Defensins: Linking Innate and Adaptive Immunity Through Dendritic and T Cell CCR6. *Science (1979)* **286**, (1999).

15. Carp, H. Mitochondrial N-formylmethionyl proteins as chemoattractants for neutrophils. *Journal of Experimental Medicine* **155**, 264–275 (1982).

16. Taylor, K. R. *et al.* Hyaluronan Fragments Stimulate Endothelial Recognition of Injury through TLR4. *Journal of Biological Chemistry* **279**, 17079–17084 (2004).

17. Johnson, G. B., Brunn, G. J., Kodaira, Y. & Platt, J. L. Receptor-Mediated Monitoring of Tissue Well-Being Via Detection of Soluble Heparan Sulfate by Toll-Like Receptor 4. *The Journal of Immunology* **168**, 5233–5239 (2002).

18. Smiley, S. T., King, J. A. & Hancock, W. W. Fibrinogen Stimulates Macrophage Chemokine Secretion Through Toll-Like Receptor 4. *The Journal of Immunology* **167**, 2887–2894 (2001).

19. Weathington, N. M. *et al.* A novel peptide CXCR ligand derived from extracellular matrix degradation during airway inflammation. *Nat Med* **12**, 317–323 (2006).

20. Brand, U. *et al.* Influence of extracellular matrix proteins on the development of cultured human dendritic cells. *Eur J Immunol* **28**, 1673–1680 (1998).

21. Houghton, A. M. *et al.* Elastin fragments drive disease progression in a murine model of emphysema -- Houghton et al_ 116 (3) 753 -- Journal of Clinical Investigation. *Journal of Clinical Investigation* **116**, 753 (2006).

22. Adair-Kirk, T. L. *et al.* A Site on Laminin α5, AQARSAASKVKVSMKF, Induces Inflammatory Cell Production of Matrix Metalloproteinase-9 and Chemotaxis. *The Journal of Immunology* **171**, 398–406 (2003).

23. Jong, W. C. *et al.* Peroxiredoxin-I is an autoimmunogenic tumor antigen in non-small cell lung cancer. *FEBS Lett* **579**, 2873–2877 (2005).

24. Iwata, Y. *et al.* Autoantibody against peroxiredoxin I, an antioxidant enzyme, in patients with systemic sclerosis: Possible association with oxidative stress. *Rheumatology* **46**, 790–795 (2007).

25. Karasawa, R., Ozaki, S., Nishioka, K. & Kato, T. Autoantibodies to peroxiredoxin I and IV in patients with systemic autoimmune diseases. *Microbiol Immunol* **49**, 57–65 (2005).

26. Xu, Y., Harder, K. W., Huntington, N. D., Hibbs, M. L. & Tarlinton, D. M. Lyn tyrosine kinase: Accentuating the positive and the negative. *Immunity* **22**, 9–18 (2005).

27. Terrier, B. *et al.* Alpha-enolase: A target of antibodies in infectious and autoimmune diseases. *Autoimmun Rev* **6**, 176–182 (2007).

28. Bogdanos, D. P. *et al.* Antibodies to soluble liver antigen and α-enolase in patients with autoimmune hepatitis. *J Autoimmune Dis* **1**, 1–6 (2004).

29. Xiang, Y. *et al.* Proteomic Surveillance of Autoimmunity in Osteoarthritis: Identification of Triosephosphate Isomerase as an Autoantigen in Patients with Osteoarthritis. *Arthritis Rheum* **50**, 1511–1521 (2004).
